# The scale of Nigeria's involvement in the trans-national illegal pangolin trade: Temporal and spatial patterns and the effectiveness of wildlife trade regulations

**DOI:** 10.1016/j.biocon.2021.109365

**Published:** 2021-12

**Authors:** Charles A. Emogor, Daniel J. Ingram, Lauren Coad, Thomas A. Worthington, Andrew Dunn, Inaoyom Imong, Andrew Balmford

**Affiliations:** a Conservation Science Group, Department of Zoology, University of Cambridge, Cambridge CB2 3QZ, UK; b IUCN SSC Pangolin Specialist Group, Zoological Society of London, London NW1 4RY, UK; c Wildlife Conservation Society, Nigeria Program, GPO Box 796, Calabar, Nigeria; d African Forest Ecology Group, Biological and Environmental Sciences, University of Stirling, Stirling FK9 4LA, UK; e Center for International Forestry Research, Bogor 16115, Indonesia; f Interdisciplinary Centre for Conservation Science, University of Oxford, Oxford OX1 3SZ, UK

**Keywords:** CITES, Illegal wildlife trade, Pholidota, Trafficking, Wildlife trafficking, African pangolins, COVID-19

## Abstract

The Convention on International Trade in Endangered Species of Wild Fauna and Flora (CITES) prohibits commercial trans-national trade in pangolin specimens. However, African pangolins are continually trafficked to Asia for traditional medicine, with Nigeria considered a key hub. Using reported Nigeria-linked pangolin seizure data and interviews with Nigerian law enforcement officials, we a) characterised Nigeria's involvement in global pangolin trafficking January 2010–September 2021, particularly observing trafficking trends after pangolin's CITES Appendix I listing; b) estimated the minimum number of pangolins whose scales are in Nigeria-linked seizures January 2010–September 2021, and; c) assessed ongoing efforts within Nigeria to curb pangolin trafficking. Nigeria-linked seizures involved 190,407 kg of pangolin derivatives (99.9% scales) from a minimum of 799,343 pangolins (95% confidence interval; 625,944-996,353) of four species (see caveats in Methods). All shipments confiscated in transit were destined for Asia, with a rapid increase in the mass of maritime shipments over time. Furthermore, stockpiling of pangolin derivatives for overseas shipment is perhaps a prominent trafficking model in Nigeria. Nigeria's law enforcement efforts improved from 2017, the same year Nigeria apparently began playing a hub role. The impact of pangolin's CITES Appendix I listing on pangolin trafficking was unclear, as the marked rise in seizures from 2017 when the listing became effective, coincided with improvements in Nigerian law enforcement efforts. COVID-19–induced travel restrictions likely reduced trafficking activities in 2020 but activities may have fully resumed in 2021. This study provides new information to inform effective enforcement and policy formulation efforts to protect African pangolins.

## Highlights

• We characterised Nigeria-linked pangolin trafficking using three types of data.
• Nigeria-linked seizures (2010-Sept 2021) involved 190,407 kg of pangolin specimens.
• These seizures contained scales from a minimum of ~0.8 million pangolins.
• CITES Appendix I listing of pangolins may not have reduced trafficking.
• Combining multiple data types enhances illegal wildlife trade research.

## 1 Introduction

Illegal wildlife trade (or wildlife trafficking) is poorly monitored and regulated (Phelps and Webb, 2015), mainly because of its clandestine, dynamic, and multifaceted nature (Phelps et al., 2016; 't Sas-Rolfes et al., 2019). This is despite substantial investments in countering the trade; in just six years, at least US$1.3 billion was invested in wildlife trafficking interventions such as community-based programmes, law enforcement, and policy development in Africa and Asia (World Bank, 2016). Illegal wildlife trade diminishes wildlife populations across different taxa (Margulies et al., 2019; Scheffers et al., 2019), threatens the livelihoods of local communities that depend on wild resources (Nielsen et al., 2018), endangers public health via the emergence of zoonoses such as COVID-19 (Haider et al., 2020), and undermines the rule of law through organised criminal networks and institutional corruption (Milliken and Shaw, 2012; Wasser et al., 2015; Viollaz et al., 2018; Wyatt et al., 2018). Pangolins (Pholidota: Manidae) are labelled the world's most trafficked wild mammals (Challender et al., 2014), with one estimate suggesting that one million individuals were traded globally in 13 years (Heinrich et al., 2017). Pangolins are only found in Africa and Asia, with the eight species split equally between the two continents. All pangolin species are listed as Vulnerable, Endangered or Critically Endangered on the Red List of Threatened Species™ (IUCN, 2021). Threats include habitat loss, local harvest for food and medicine, and electrocution on fences (for *Smutsia temminckii*). The rising demand for their scales, a widely used constituent in traditional Chinese medicines (Wang et al., 2020; Zhang, 2009), has dramatically increased their exploitation in the last decade (Heinrich et al., 2017). As Asian pangolin populations have declined (Chong et al., 2020; Mahmood et al., 2020; Schoppe et al., 2020; Wu et al., 2020), their African congeners have faced increasing pressure to meet Asia's demand for pangolin scales, and to a lesser extent, meat (Challender et al., 2020; Ingram et al., 2019).

Nigeria, home to three of the four African pangolin species, has been identified as a major transit country for trafficking wildlife products – especially pangolin scales and ivory – between Africa and Asia (Gomez and Leupen, 2016; Omifolaji et al., 2020; UNODC, 2020b). Nigeria is Party to the Convention on International Trade in Endangered Species of Wild Fauna and Flora (CITES), which, through its Appendix I listing (effective from January 2017), prohibits international commercial trade of wild-caught pangolins and their derivatives. Additionally, Nigeria's wildlife harvest and trade legislation, the Endangered Species Act No 11 of 1985 (amended in 2016) list pangolins under Schedule I, prohibiting “the hunting or capture of or trade” in pangolins (Endangered Species (Control of International Trade and Traffic) Act, 1985, p1). Contravention of the Act by hunting, possessing, or trading in pangolins attracts a fine of NGN5,000,000 (approximately US$12,150; at US$1 = NGN411) – revised from NGN1,000 stated in the 1985 version of the Act – for the first offence, and one-year imprisonment without the option of a fine for the second and subsequent offences (Endangered Species (Control of International Trade and Traffic) (Amendment) Act, 2016). However, despite these regulations, Nigeria has been involved in more reported pangolin trafficking incidents than any other African country (Heinrich et al., 2017).

A comprehensive assessment of Nigeria's involvement in pangolin trafficking is thus important for informing effective law enforcement and policy development efforts for pangolins and numerous other wild species also threatened by trans-national illegal trade. Previous studies on Nigeria's preeminent role in pangolin trafficking (Gomez and Leupen, 2016; Omifolaji et al., 2020) were either limited in scope (for example; not explicitly quantifying pangolin trade linked to Nigeria) or temporal scale (i.e., omitting reported trafficking incidents in 2010 when the first pangolin seizure linked to Nigeria was documented). In this study, we combine quantitative and qualitative analyses to characterise and quantify Nigeria's role in pangolin trafficking from January 2010 to September 2021. We asked the following research questions. First, what is the modus operandi of pangolin trafficking linked to Nigeria – including the quantity of seized pangolins and their derivatives (meat, scales and claws, and skin; hereafter pangolin derivatives), their trafficking networks and routes, key trafficking locations, and detection methods used by law enforcement agencies? Second, what is the minimum number of pangolins involved in the reported Nigeria-linked pangolin seizure? Third, what are the predictors of trafficked shipment mass of pangolin derivatives for reported Nigeria-linked seizures from 2010 to 2020? Fourth, how did pangolin CITES Appendix I listing impact Nigeria-linked pangolin trafficking? Additionally, we briefly explored the impacts of COVID-19–induced international travel restrictions on pangolin trafficking, hypothesising that COVID-19–induced international travel restrictions halted possible trafficking of pangolin derivatives in and out of Nigeria (see Aditya et al., 2021).

## 2 Materials and methods

We used three inter-related but distinct data types: a) records of reported pangolin seizure events linked to Nigeria, occurring within and outside the country; b) mass measurements of confiscated pangolin scales, and; c) semi-structured interviews with law enforcement officials in Nigeria.

### 2.1 Collection and curation of seizure records

We obtained pangolin seizure records from January 2010 to September 2021 from TRAFFIC's Wildlife Trade Portal (available at https://www.wildlifetradeportal.org; accessed on 30 September 2021), and collected pangolin seizure datasets for the same period from Wildlife Conservation Society's Counter Wildlife Trafficking Program, Wildlife Justice Commission, Environmental Investigation Agency and Nigeria Customs Service (NCS). We aggregated datasets from these sources to maximise the coverage of reported seizures linked to Nigeria. Except for data from NCS that were exclusively records of wildlife seizures made within Nigeria by NCS staff, seizure records from other databases contained manually curated open-source records, with the majority from media publications, government press releases, court verdicts, and CITES reports. To avoid double-counting, we used TRAFFIC's database as a reference, comparing other data sources against it to identify unique seizures based on information for each record such as seizure date, number or mass of items seized, and seizure location (site and country). We were attentive to inconsistencies and possible misreporting, paying special attention to the reported mass of seizures and the measurement unit used. We observed approximately 90% similarity between databases (except for incidents involving <500 kg of derivatives intentionally excluded from Wildlife Justice Commission's database), with at least two of the four databases containing any one seizure record, excluding NCS database that contained two unique seizures made in 2018. Reported masses of seizures (including reported units of measurement) comprised the most inconsistent information across databases (approximately 20% of total records). However, we addressed inconsistencies in seizure mass through an internet search of associated weblinks included in the databases (using https://web.archive.org/ to retrieve archived weblinks), finally selecting the lowest reported mass, if our internet search for the original report was unsuccessful. Where possible, for each seizure incident, we recorded the mass, location of the seizure incident, trade routes, transport mode or seizure location (when not associated with any transport mode), and whether suspects were arrested or prosecuted when confiscations were made. We defined the mode of transport for each shipment as the transport method used for the longest portion of the trade route. We classed seizures with undocumented transport mode as ‘unknown’, and grouped confiscations made in residential or business premises without information on the origin and destination of the materials as ‘warehouse’ seizures. We used this information to create a new seizure database which we used for further analyses.

#### 2.1.1 Seizure records analysis

We quantified differences in seizure mass across transport modes using a Kruskal-Wallis test and Dunn's post-hoc test. We also tested for possible relationships between shipment mass, shipment year, and transport mode, but restricted this analysis to 2010–2020 as including the incomplete 2021 data (<12 months) would skew our results. We used a generalised linear model with mass as the response variable (log_10_-transformed to meet the assumption of residual normality) and mode and year (fitted as a continuous variable) as predictor variables, back-transforming the data to visualise the results. We assessed different combinations of the variables, and using the R package *AICcmodavg* (Mazerolle, 2020), we compared their Akaike Information Criterion (AIC) following Aho et al. (2014), and selected the model (inclusive of interaction terms) which had the lowest AIC.

Our database contained 80 individual records, representing either the seizure of pangolin derivatives from a single location within a country or the movement of pangolin derivates within or between countries. This data allowed us to identify trade routes to determine the role of counties in the movement of pangolin derivates. For each record, implicated countries were classified into one of three roles: the shipment origin, a transit country, or the import country. For records where the data solely identified a seizure from a single location within a country, that country was classified as the shipment origin. Where the record identified movements within or between countries, the country of first location recorded was classified as the shipment origin, with the country of the final location classified as the import country. If an individual record contained three or more locations, the countries of the intermediate locations were classified as transit countries. Using a ternary plot constructed using the *tricolore* package in R (Schöley and Kashnitsky, 2020), we then assessed pangolin trade routes for a set of intervals from 2010 to 2021 (2010–12; 2013–15; 2016–18; 2019–September 2021). We excluded confiscations classed as ‘unknown’ and ‘warehouse’ from the flow map analysis. We explored trends in trafficking before and after the CITES ban on international commercial trade on all pangolin species. Although the amendment proposals to list all pangolin species under Appendix I was adopted by CITES in October 2016, the ban only became effective in January 2017 (CITES, 2016a, CITES, 2016b). Data analyses were conducted in R version 1.3.959 (R Core Team, 2021).

### 2.2 Estimation of the minimum number of pangolin individuals

To better understand the extent of Nigeria's involvement in global pangolin trafficking, we attempted to quantify the minimum number of individuals (MNI) that contributed to the seized shipment. We defined MNI as the smallest possible number of individual pangolins (across all species) needed to produce a unit mass of trafficked scales, assuming all scales on each animal were used. The scales (stored in sacks) in the custody of NCS and National Environmental Standards and Regulations Enforcement Agency (NESREA) are believed to be the bulk of the pangolin seizures made by the NCS since 2010 when the first record of confiscation in Nigeria became available. Usually, when wildlife materials are confiscated by law enforcement agencies such as NCS, they are handed to NESREA, the agency responsible for enforcing environmental standards and laws in Nigeria. NESREA is then expected to document seizures, conduct a further investigation (where appropriate) and prosecute suspected traffickers (if apprehended).

#### 2.2.1 Data collection and analysis for MNI estimation

**Fig. 1:**
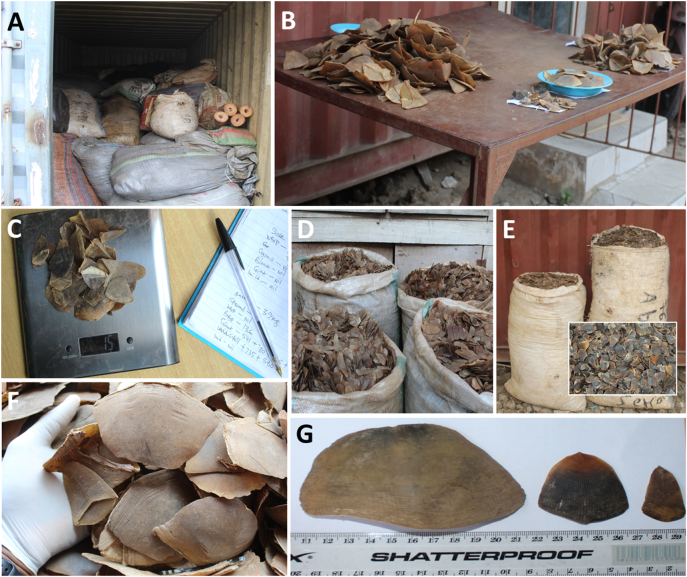
Selection of confiscated scales and illustration of the scale-sorting process. One of the sampled warehouses containing pangolin scales with other confiscated wildlife products (ivory; A). Handfuls of scales (giant ground pangolins in the photo) scooped from sacks onto a platform for sorting (B). Weighing and recording of scale mass (15 g of white-bellied pangolin scales in the photo; C). Sacks containing scales from only one species; white-bellied pangolin (D) and black-bellied pangolin (E). Sorted scales belonging to giant ground pangolins (F). Examples of dorsal scale (nape to base of tail) for each species group (left to right: *Smutsia* spp., black-bellied pangolin, white-bellied pangolin; G).

We estimated pangolin MNI from reported Nigeria-linked seizures from January 2010 to September 2021 in two broad steps. We first assessed the proportion of each of the four African pangolin species in pangolin seizures impounded in Nigeria. Secondly, we applied the mean Mass-to-Individual Conversion Factors (dried mass of pangolin scale and claws per individual; Appendix A) of each species to the obtained proportions and total scale mass of seizures linked to Nigeria from 2010 to September 2021. To obtain the proportion of each African pangolin species in reported seizures, we sampled confiscated pangolin scales stored in four warehouses across two locations in Nigeria (one in Abuja and three in Lagos). These sampled confiscated scales represent different seizure incidents, including those we labelled ‘warehouse’ and those trafficked by any of the transport modes (we did not sample one remaining warehouse because of logistical constraints). To obtain a sample of the seized pangolin scales, we first haphazardly selected the sampled sacks because the stacking of sacks in some warehouses (e.g., in Fig. 1A) hindered efforts to sample them randomly. However, we made sure to sample sacks that were covered by other sacks and those of different packaging and size. Where possible, we counted and recorded the total number of sacks and recorded the date of seizures, trade route, and the mass trafficked (if known). We then a) with two hands, scooped five handfuls from the top of the sack (Fig. 1B); b) emptied the remaining content of the sack into a large container, and; c) scooped another five handfuls from the bottom of the sack, adding it to the initial five handfuls (i.e., ten handfuls per sack). Next, we identified the species to which each scale belonged, weighed the sorted scale and recorded their mass(es) against the mass of the sampled sack (Fig. 1C). Using Cota-Larson (2017) as a guide, we carefully examined each scale visually, differentiating them by colour, shape and/or size into a) black-bellied pangolin (*Phataginus tetradactyla*); b) giant ground pangolin (*Smutsia gigantea*); c) Temminck's pangolin (*Smutsia temminckii*); d) white-bellied pangolin (*Phataginus tricuspis*), and; e) unidentified pangolin. We sampled 67 sacks ranging from 6 to 104 kg, some of which contained scales from a single species (Fig. 1D & E). Rotten or degraded scales were not sorted and unless attached, we identified and recorded each scale independently (claws were mostly attached to scales). We were confident in our identification of *P. tetradactyla* and *P. tricuspis* but found it difficult to differentiate between *S. gigantea* and *S. temminckii*. We thus aggregated scale masses from the latter pair as *Smutsia* spp. (Fig. 1F) and used the mean of their Mass-to-Individual Conversion Factors for further analyses. We derived MNI estimates of each category (i.e., *P. tetradactyla*, *Smutsia* spp. and *P. tricuspis*; Fig. 1G) as follows. First, we calculated the relative proportional mass of sample scales (*p*_*rm*_) for each species group within each of the 67 sacks. Second, for each group, we used the R package *boot* (Canty and Ripley, 2021; Carpenter, 1998) to estimate the mean and 95% confidence intervals (CI) of *p*_*rm*_ using 1000 bootstrapped replicates and the adjusted bootstrap percentile (BCa) method, which accounts for possible skewness and bias in the data we analysed (Puth et al., 2015). Third, we converted the bootstrapped means and 95% CI of *p*_*rm*_ to MNIs for each group by multiplying the total confiscated scale mass from our seizure records (*m*_*cs*_) and dividing by Mass-to-Individual Conversion Factors (*CF*_*M*→*I*_) using the following equation:

$$\frac{p_{\mathrm{rm}}\times m_{\mathrm{cs}}}{{\mathrm{CF}}_{M\to I}}$$

#### 2.2.2 Caveats and assumptions of MNI estimation method

Our MNI estimate involved three main assumptions. First, we assumed that the relative proportional mass of sampled scales is representative of the relative proportional mass of all confiscated scales in seizures linked to Nigeria across our study period. Second, we assumed that all scales on animals contributing to the assessed shipments were contained in those seizures (i.e., all scales from the pangolins in seized shipments were removed and included in the shipment, and none were discarded before being shipped). Lastly, we assumed that the mean Mass-to-Individual Conversion Factors are appropriate for the animals whose scales are in the trade. We acknowledge the variation in, and small sample size of Mass-to-Individual Conversion Factors especially for black- and white-bellied pangolins (n = 6 and n = 7, respectively) which could influence our MNI estimates. Note that as conversion factors from the same site (country) are more comparable, but less comparable to those from other sites, these variations may account for geographic influences in pangolin morphology, increasing the representativeness of our samples.

### 2.3 Semi-structured interviews

In order to characterise efforts to curb pangolin trafficking in Nigeria, we conducted semi-structured interviews (Newing et al., 2010) with ten NCS staff in August 2020. The interviews focused on a) trends in enforcement efforts of NCS to curb pangolin trafficking; b) levels of awareness of pangolin trafficking within NCS, and; c) outcomes of detected cases of pangolin trafficking. We used semi-structured interviews as these allowed us to gather in-depth information on the focal interview topics. Respondents from different locations across Nigeria were recommended by the Regional Intelligence Liaison Office of NCS. However, no participant was informed of the focal interview topics before the interview. Nonetheless, all the participants know about pangolin trafficking and are, to an extent, actively involved in curbing the illicit trade. Since our analyses of the seizure data focused on determining any changes from 2017 when the ban on commercial international trade of pangolins became effective, we designed our interview questions to address two periods: a) before 2017, and; b) from 2017. We recorded the interviews and checked for within-individual consistency by comparing responses to related questions. We obtained ethics approval for this study from the University of Cambridge’s Psychology Research Ethics Committee (application number: PRE.2020.095).

## 3 Results

### 3.1 Pangolin seizures linked to Nigeria

We identified 80 Nigeria-linked seizures from January 2010 to September 2021 (Supplementary material). Three seizures lacked mass data, so we restricted our analyses to 77 seizures totalling 190,407 kg. Scales consisted of 190,404 kg with one dried carcass (meat) accounting for the remaining 3 kg. The records without mass data were a) a wallet made from pangolin scales seized in Nigeria in 2010; b) a dried pangolin carcass seized in Nigeria in 2011, and; c) 23 sacks of pangolin scales destined for air shipment from Nigeria to China, detected in Nigeria in 2018. A total of 18,412 kg of ivory was found in 26 of the seizures (with an unreported mass of ivory found in three others). African grey parrots (*Psittacus erithacus*; 124 dried carcasses), agarwood (4 kg), khat (*Catha edulis*; 300 kg), unidentified shark fin (61.3 kg), worked coral stones, rhino horn, unidentified donkey (Equidae) skin, crocodile (Crocodylinae) skin, and unidentified animal bones were also discovered.

**Fig. 2:**
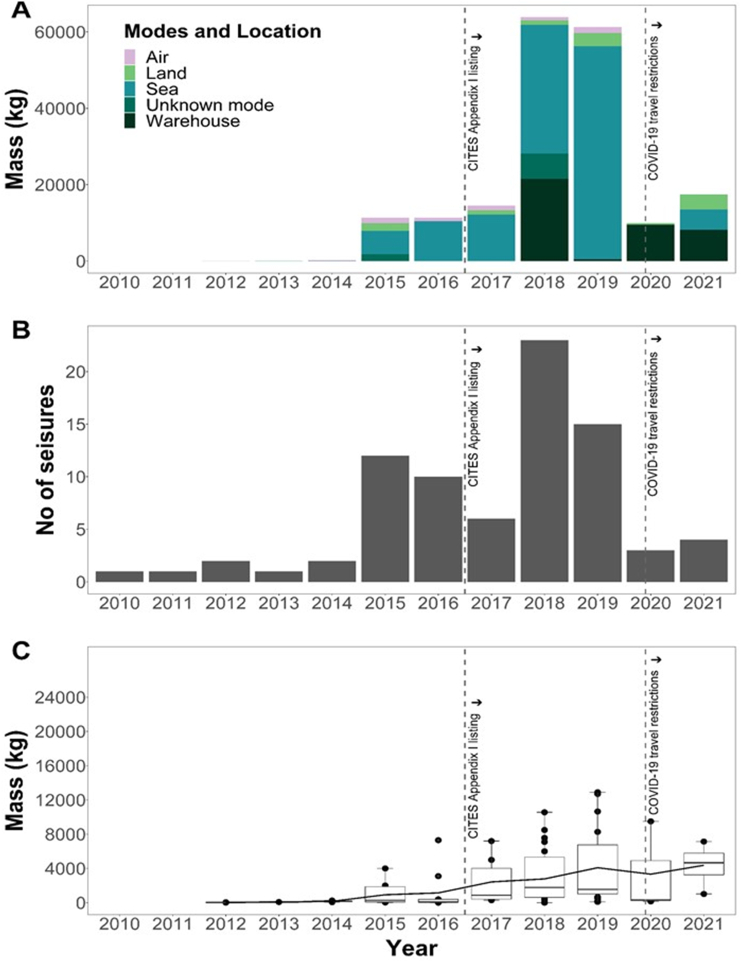
Yearly reported confiscated mass of seizures linked to Nigeria from January 2010 to September 2021 showing the associated modes of transport or location of pangolin derivatives when confiscated (A). Yearly reported number of confiscations linked to Nigeria from January 2010 to September 2021 (B). The mean mass per seizure linked to Nigeria from January 2010 to September 2021 (line; left y-axis), and a boxplot showing confiscated masses of each seizure event (black dots), median mass (horizontal line in each box), and largest and smallest masses for each year (whiskers) (C). From left to right, vertical dashed lines indicate a) when CITES ban on commercial international trade was brought into effect (January 2017), and; b) when global COVID-19 travel restrictions were made (approximately April 2020). Mass data were not included in panels B and C for 2011 and 2012 (count data were available for these years). Data for 2021 ends in September.

The annual total mass of trafficked pangolin derivatives varied considerably by year and transport mode (or location when confiscated), with sharp increases in 2018 and 2019 (Fig. 2A). Pangolin derivatives were trafficked via air, land, and sea but pangolin derivatives trafficked via sea made up 65% of the total mass seized (123,636 kg; Fig. 2A). From 2018, confiscations were not limited to transported consignments, but seizures included pangolin derivatives stored in warehouses (Fig. 2A), all of which were seized by NCS in Nigeria (39,726 kg from seven seizures). The remaining eight seizures made within Nigeria from 2018 involved air, land, and sea transports, accounting for 8310 kg of seized trafficked mass (plus 23 sacks lacking mass data). The number of reported seizures also varied considerably over time, with less than five seizures each year before 2015, rising to 22 confiscations in 2018, followed by fewer reported seizures in 2019 to September 2021 (Fig. 2B). Mean reported seizure mass increased steadily from 2012 to 2019 and then apparently fell in 2020 before rising again in 2021 (Fig. 2C).

**Fig. 3:**
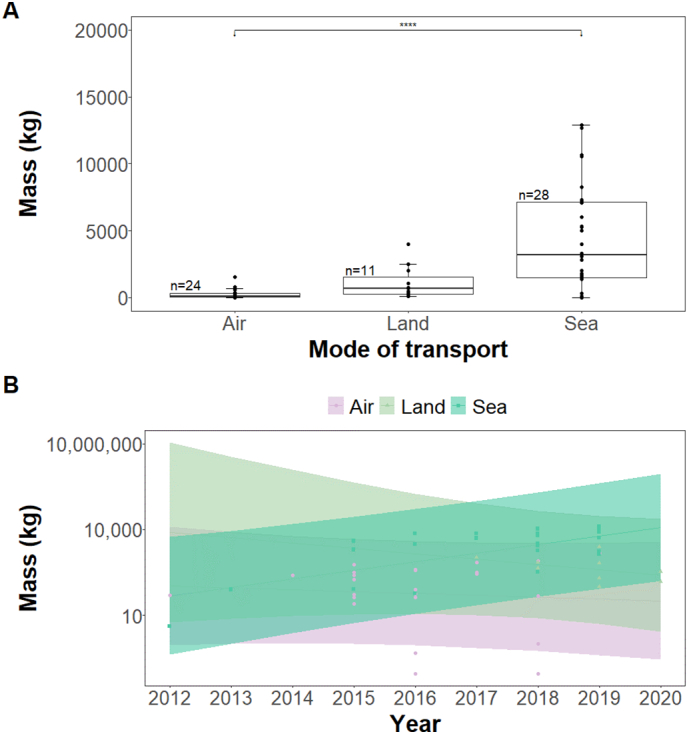
Difference in the mass of reported Nigeria-linked pangolin derivatives trafficked via air, land, and sea from January 2010 to September 2021 (A), and how these change from 2010 to 2020 (B). In A, n is the number of seizures for each mode; the horizontal line in each boxplot represents the median masses for the associated modes; whiskers are the minimum and maximum masses. The horizontal line above the boxplots shows the comparison between air and sea transports, with the asterisks indicating a significant difference in the masses trafficked by both modes. In B, the shaded parts on either side of the regression line are the 95% confidence intervals. Seizures with unknown transport modes and those confiscated from warehouses were not included in the model and thus not presented.

The mass of pangolin derivatives per seizure differed with mode of transport and was greatest for those transported by sea and lowest for those trafficked by air (Kruskal-Wallis test: χ^2^ = 28.3, df = 2; n = 53; *P* < 0.001; post-hoc Dunn's tests, sea vs air: *P* < 0.001; sea vs land: *P* = 0.03; land vs air: *P* = 0.01; Fig. 3A). The generalised linear model (R^2^ = 0.42, F_5,55_ = 9.73, *P* < 0.001; Table 1) indicated that seizure mass changed over the years and between transport mode, with masses of consignments trafficked by sea increasing as masses trafficked via air and land declined (Fig. 3B).

**Table 1:** Regression model predicting variations in the mass of reported Nigeria-linked pangolin seizures in relation to year (2010–2020) and transport modes.

Variables	Coefficient estimate	Standard error (SE)	Beta coefficient (β)	*P*-value (*P*)
Intercept	132.35	241.74	0.00	0.59
Year	‐0.06	0.12	‐0.11	0.59
Land	249.31	465.02	79.65	0.59
Sea	‐731.40	302.29	‐313.49	0.02
Year: Land	‐0.12	0.23	‐79.30	0.60
Year: Sea	0.36	0.15	314.17	0.02

**Fig. 4:**
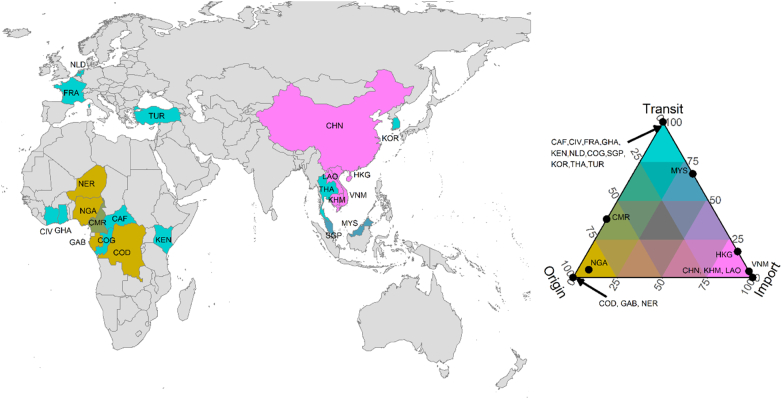
Countries implicated in the global illegal pangolin trade linked to Nigeria. Countries were classed into origin (amber), transit (cyan), and import (pink) roles. The legend describes the roles of countries (identifiable by their 3-digit ISO codes). Dots on corners represent countries that were exclusively associated with a single role. Dots on the edge of the plot represent countries that played dual roles (for example, Vietnam played 75% import and 25% transit roles) while the dot inside the triangle represents the only country (Nigeria in this case) that played all three roles (albeit acting largely as a source country). (For interpretation of the references to colour in this figure legend, the reader is referred to the web version of this article.)

Reported global illegal pangolin trade linked to Nigeria from January 2010 to September 2021 involved 21 other countries or territories (nine in Africa, nine in Asia, and three in Europe; Fig. 4). All the implicated African countries played either origin (Democratic Republic of the Congo, Gabon, and Niger) or transit roles (except Nigeria that also played import roles). All confiscations (except warehouse seizures) were destined for Asia, with European countries – France, the Netherlands and Turkey – transit locations for shipments leaving Africa for Asia. Several other countries played transit roles, but China, Cambodia, and Lao People's Democratic Republic were recorded solely as import countries (Fig. 4).

**Fig. 5:**
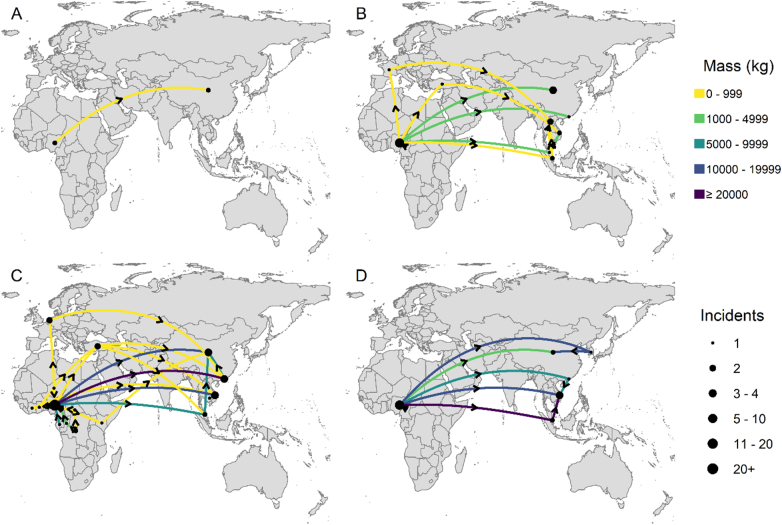
Flow maps of the global illegal pangolin trade involving Nigeria for 2010–2012 (A), 2013–2015 (B), 2016–2018 (C) and 2019–September 2020 (D).

The first reported Nigeria-linked pangolin seizure confiscated outside Nigeria was in China in May 2012. In the same year, another shipment from Nigeria was also confiscated in China; the combined seizures weighed 55 kg (Fig. 5A). In 2013, Nigeria played a transit role for a shipment to China (80 kg) that originated from Cameroon. Nigeria then became the sole source of the 11,661 kg of Nigeria-linked seizures from then until 2015 (Fig. 5B). The hub role Nigeria played became apparent in 2017 (Fig. 5C) when Nigeria received two seized shipments from Cameroon and one seized shipment from Gabon collectively weighing 6754 kg. In the following years, Nigeria acted as a connection for shipments destined for Asia, with seized consignments originating from the Democratic Republic of Congo, Cameroon, Republic of Congo, Niger, and the Central African Republic. Some of these shipments lacked information on the next phase of the journey and were reported with Nigeria as an import country. However, pangolin derivatives also left Nigeria to other African countries before finally leaving the continent for Asia. For example, 0.5 kg was transported via air from Nigeria through Kenya to China in 2016; 288 kg left Nigeria via air to Malaysia through Ghana and Turkey, and 600 kg originating from Côte d'Ivoire was routed through Nigeria to Vietnam (unreported transport mode). From 2019, all reported seized shipments (n = 22) originated from Nigeria, except two shipments totalling 6500 kg that were destined to Nigeria from Cameroon (Fig. 5D). All the Nigeria-linked confiscations in 2020 (n = 3) and three of four of 2021 seizures were made in Nigeria. Approximately 77% (138,430 kg) of the total detected mass were destined for Asia (the bulk of the remainder were warehouse seizures in Nigeria). The highest quantities of pangolin scales destined for any country or territory were Vietnam (64,039 kg; n = 18), China (48,364 kg; n = 20), and Hong Kong (21,279 kg; n = 10).

At least 67% of the mass and number of seizures between January 2010 and September 2021 that were informed by intelligence-gathering (66,384 kg; 14 seizures) were made in Nigeria (Table 2), most of which were seized from warehouses (39,727 kg of the 46,559 kg seized within Nigeria). China and Hong Kong also relied on intelligence-gathering to intercept shipments. However, for seized shipments involving Nigeria, Hong Kong intercepted 28,200 kg of pangolin derivatives through routine inspection; China and Nigeria intercepted only 62 kg and 1503 kg, respectively, through this method. Inspection using scanners led to the confiscation of 903 kg of pangolin materials in three Asian countries (Table 2). Port Harcourt international airport, Mallam Aminu Kano international airport and Lagos' Apapa seaport were the only Nigerian ports linked with a single pangolin trafficking incident of over 500 kg. More of the seized shipments (by mass) were trafficked through ports linked to Hong Kong's Kwai Chung customhouse cargo examination compound and Tsing Yi cargo examination compound than any other port (Appendix B). The 27 seizure incidents that occurred in Nigeria involved 44 suspects. A total of 20 suspects were arrested in connection to the seizures in Nigeria, with four suspects facing prosecutions (the enforcement and legal outcomes of incidents involving the remaining suspects are unknown; Appendix C).

**Table 2:** Detection methods used by law enforcement officials in discovering and confiscating trafficked pangolin derivatives linked to Nigeria from January 2010 to September 2021. Information on detection methods was only available for 41 incidents (~50% of the total number of confiscations).

Incident country	Detection method	Mass (kg)	Number of incidents
Kenya	Sniffer dogs	0.5	1
Cameroon	Intelligence	2500	1
Hong Kong	Intelligence	8268	1
China	Intelligence	10,560	1
Nigeria	Intelligence	45,056	11
Cameroon	Routine inspection	5050	2
China	Routine inspection	62	2
France	Routine inspection	250	1
Hong Kong	Routine inspection	28,200	7
Singapore	Routine inspection	13,024	2
Vietnam	Routine inspection	10,624	3
Nigeria	Routine inspection	1503	4
Vietnam	Scanner	42	1
China	Scanner	274	2
Thailand	Scanner	587	2
**Total**	**–**	**126,000.5**	**41**

### 3.2 Minimum number of pangolin individuals involved in Nigeria-linked trafficking from 2010 to September 2021

We identified scales belonging to all African pangolin species in the 67 sampled sacks (totalling 2475 kg) – although we grouped giant ground and Temminck's pangolins to increase the confidence of our estimates. White-bellied pangolin scales were the most numerous, with 43 sacks containing scales from only this species. A small number of sampled sacks (n = 3) also contained scales from only black-bellied pangolins, while the remainder (n = 21) contained scales from at least two pangolin species (including black- and white-bellied pangolins in some cases). Of the 291 kg of pangolin scales we identified to species, white-bellied pangolins represented 71.4%; giant ground and Temminck's pangolins collectively accounted for 17.6%, while black-bellied pangolins and unidentified scales made up 10.7% and 0.3%, respectively. We estimated that seizures from the global illegal pangolin trade linked to Nigeria from 2010 to September 2021 consisted of scales from at least 799,300 pangolins (95% confidence interval; 625,900-996,300; Table 3).

**Table 3:** Estimated equivalent minimum number of pangolins trafficked for seizures made from January 2010 to September 2020, based on pangolin scales in reported Nigeria-linked seizures. Note that 876 g of scales were unidentified.

Species category	Sorted mass (kg)	Estimated mass in seizures (kg) and 95% confidence interval limits			MNI in seizures and 95% confidence interval limits			% of MNI
		Mass	Lower limit	Upper limit	MNI	Lower limit	Upper limit	
White- bellied pangolin	207.86	127,197.3	103,571.1	146,752.1	717,090	583,894	827,332	89.71
Black-bellied pangolin	31.17	13,015.5	6103.43	28,710.82	65,434	30,684	144,341	8.19
*Smutsia* spp.	7.96	49,362.75	33,360.19	72,435.52	16,819	11,366	24,680	2.10
**Total**	**246.99**	**189,576**	**143,035**	**247,358**	**799,343**	**625,944**	**996,353**	**100**

### 3.3 Enforcement personnel interviews

Our interviews with ten NCS staff members revealed that the number of officials conducting inspections depends on the traffic at the seaport or land border, with relatively more officials (approximately ten teams of 9–12 inspectors) inspecting containers at busy seaports such as Apapa and Tin Can Island which receive approximately 500 containers daily. Average-traffic seaports and land borders deal with a daily flow of 100–150 containers and 60–100 trucks, respectively, while Lagos' Murtala Muhammed International Airport (LOS) processes approximately 4000 average-sized parcel pallets weekly. Respondents reported that 70–90% of all exported and collected imported containers are examined, but with only cursory inspections involving cutting open the seals and visually inspecting exposed parts of the container's content. The daily inspection rate at the land borders – before their closure in 2012 to curb illegal importation of goods – was approximately 30% of the vehicles received daily. The daily inspection rate was slightly higher at seaports (50%) but was non-existent for parcels leaving Nigeria through LOS (Nigeria's busiest airport) until 2012. Only one respondent was directly involved in intercepting pangolin scales leaving Nigeria while two respondents were involved in intelligence-gathering operations that resulted in pangolin seizures within Nigeria. Respondents reported that intelligence-gathering has been pivotal in the recent Nigerian seizures (2018–2020) and thus has been prioritised, especially as the automated container scanners reported to “more than double physical examination speed”, have been non-functional since 2018. Although traffickers were rarely arrested during confiscations (number of respondents = 6), arrested suspects were transferred to NESREA (interviewees were unaware of any pangolin prosecutions, reporting that most cases of arrested traffickers were ‘settled out of court’; these reports corroborate seizure data information outlined in Appendix C).

All respondents reported to have participated in at least two Illegal Wildlife Trade (IWT) training courses. Assessing the situation over time, respondents said that on average, the number of training opportunities aimed at increasing the detection and interception of illegally traded wildlife derivatives stayed the same from 2011 to 2016 with only very few training courses held. However, training opportunities later increased from 2017 to 2020 (approximately 51% increase compared to 2011–2016) with organisations including World Customs Organisation, Nigeria Program of Wildlife Conservation Society, and US Fish and Wildlife Service organising periodic training courses and workshops. Conversely, the number of staff deployed to inspect containers from 2011 to 2016 increased and then stayed the same from 2017 to 2020. The number of container searches increased from 2011 to 2016 and increased further in later years. Note that respondents were unable to estimate the number of staff deployed to search containers and the number of container searches conducted. Their suggestions were based on factors such as periods of mass recruitments and rate of mass deployment of officials to ports. There was uncertainty about whether what happens to arrested suspects changed from 2011 to 2016 and 2017 to 2020. Nonetheless, one respondent reported that in 2019, delegates from the World Customs Organisation revealed that NCS also has the mandate (in addition to NESREA) to prosecute wildlife traffickers in cases they uncover. The respondent, however, attributed the lack of NCS-prosecuted wildlife trafficking cases since then to the lack of arrests of suspected traffickers during confiscation. When asked about possible changes in the mode of trafficking between the two periods, respondents revealed that scales were not heavily concealed from 2011 to 2016 and that air transport was favoured more by traffickers during this period. They added, however, that trafficked masses increased from 2017, along with efforts at concealment. Respondents also reported that levels of staff awareness of pangolins trafficking (i.e., knowledge on the illegality of commercial international trade in pangolin derivatives) within NCS within NCS stayed the same – at a very low level – from 2011 to 2016 but increased markedly from 2017 to 2020. Complete interview questions are in Appendix D.

## 4 Discussion

Nigeria's involvement in the global illegal pangolin trade, probably first documented in 2012 (Challender and Hywood, 2012), has changed from the trade origin of pangolin shipments to East Asia (Gomez and Leupen, 2016) to a more complex and dynamic role of receiving and possibly stockpiling pangolin derivatives obtained from Central and other West African countries, prior to large-scale shipment to Asia. Almost all the reported seized shipments were of scales, most of which have recently been transported by sea; a change from air transport, which was more common in 2010–2015 (Gomez and Leupen, 2016; Heinrich et al., 2017). Reported Nigeria-linked seizures from January 2010 to September 2021 totalled 190,407 kg of pangolin derivatives and involved a minimum of approximately 799,300 pangolins (see Table 3). These MNI estimates are not without caveats and assumptions; particularly that the conversion factors are representative of the sampled scales and that these are in turn representative of the confiscated scales from the reported seizures used to estimate the MNI (see Section 2.2.2 for more caveats and assumptions).

Unlike in Zimbabwe where most confiscated pangolin derivatives were possibly intended for local use (Shepherd et al., 2017), all Nigeria's reported seizures were destined for overseas consumption, with high demand from Vietnam, China and Hong Kong (see also Gomez and Leupen, 2016; Heinrich et al., 2017; Ingram et al., 2019). The presence of 18,412 kg of ivory in 26 seizures makes clear there is a connection between pangolin and the longstanding ivory trafficking and hints at an organised network of pangolin traffickers often associated with ivory trafficking (see Wasser et al., 2018). The connection between pangolin and ivory trafficking corroborates findings from other countries in West and Central Africa, particularly neighbouring Cameroon (Ingram et al., 2019). The reason for Nigeria's leading role in the trade is unknown, but could be attributed to its strong infrastructure for international travel. Corruption has also been highlighted as a possible reason (Wildlife Justice Commission, 2020), with Nigeria scoring very low in the Corruption Perceptions Index in the last decade (Nigeria scored 25/100 in 2020, where 0 is very corrupt and 100 is very transparent; https://www.transparency.org/en/cpi/2020/index/nga). Furthermore, the small number of pangolin trafficking suspects prosecuted in Nigeria (n = 4 as of September 2021) over the years (Appendix C) suggests weak law enforcement (see Shepherd and Nijman, 2008). These four suspects were all prosecuted by NCS in 2021 making the first known prosecutions of pangolin trafficking suspects in Nigeria since 2010.

Asia's increasing demand for African pangolins is most likely because of the population decline of Asian pangolin populations since the late 20th century (Irshad et al., 2015; Legakul and McNeely, 1988; Newton et al., 2008; Schoppe et al., 2020; Wu et al., 2004a). The widespread use of pangolin scales in traditional medicine (Wang et al., 2020; Zhang, 2009) and the high social status associated with consuming pangolin meat (Shairp et al., 2016) in Asia are primarily responsible for Asian pangolin declines, with growing economic ties between Asia and Africa (Ajakaiye and Kaplinsky, 2009) possibly facilitating the Africa-Asia pangolin trade. Nigeria became a hub for Africa-Asia pangolin trafficking in 2017; the same year pangolin's CITES Appendix I listing became effective. This period coincides with an improvement in law enforcement efforts, inferred from the increase in the number of training courses, an increase in awareness of IWT issues within NCS, and increased international collaborations (see Section 3.2). This considerable change in law enforcement efforts thus hinders a more nuanced interpretation of possible impacts of CITES Appendix I listing on pangolin trafficking. However, the continued trafficking of derivatives from 2017 despite the ban suggests that the Appendix I listing contributed minimally to reducing the illegal trade (see Phelps et al., 2010).

We found similarities and disparities between our results and previous work on Nigeria's role in pangolin trafficking. For example, we documented 11,796 kg of derivatives trafficked between 2010 and 2015, but Gomez and Leupen (2016) reported a slightly lower mass (9715 kg) for that period. While Omifolaji et al.'s (2020) report of a total mass of 240,507 kg seized between 2011 and 2019 far exceeds the 162,936 kg we documented. However, the lack of supplementary data hindered cross-validation of these seizure data with ours, so the disparity may stem from the quality of the datasets used. For example, we did not include 3000 kg and 1331 kg of meat and scales, respectively, reportedly shipped to China from Nigeria (Gomez and Leupen, 2016; Omifolaji et al., 2020) this shipment was missing from all our datasets and we could not validate the data because the specific media webpage that apparently reported this seizure no longer works. As in previous studies, we found only a negligible mass of pangolin meat in seizures, despite its high value in import countries. This could be partly because most trafficked scales are by-products of local pangolin harvesting for food or that Asian demand for pangolin scales far exceeds the demand for meat. Traffickers may also be seeking to limit the risk of confiscation as transporting fresh or smoked meat would require cold storage and increase detection by sniffer dogs, respectively. Additionally, given meat is more perishable relative to scales, trafficking meat would restrict the possibility of stockpiling derivatives, which we suspect is part of the modus operandi of the illegal trade linked to Nigeria. Although there is no clear evidence of stockpiling within Nigeria, two shipments to Nigeria from Cameroon (5050 kg) and one from Niger (450 kg) that lacked information on the next phase of the journey suggest possible aggregation of scales prior to large-scale sea shipment. Additionally, two seizures (July and September 2021) in Nigeria contained pangolin claws (totalling 9.6 kg) that were separated from scales, presenting a new packaging method that is different from the mixing of scales and claws in previously reported Nigeria-linked pangolin seizures. This new packaging method could be because of specific overseas demand for pangolin claws (for example; for amulets made of pangolin claws in China [Wang, 2021]). Additionally, these two seizures suggest that traffickers may be dividing their consignments prior to shipping to minimise losses if shipments are seized (the seizures occured in the same city, two months apart).

Only one seizure directly linked to the sea was made in Nigeria (all other sea seizures were made in Asia except two shipments made in Cameroon and the Democratic Republic of Congo). However, the large masses associated with warehouse seizures, all of which were through intelligence-gathering operations, suggests that they were bound for sea transport. Intelligence-gathering was responsible for 45,057 kg of the 46,560 kg of derivatives seized in Nigeria from January 2010 to September 2021. The relatively small seaport seizures reported in Nigeria despite these striking warehouse confiscations thus suggests that the absence of functioning equipment may be hampering NCS law enforcement efforts at seaports. Furthermore, the absence of any reported Nigeria-linked seizures from March–September 2020, when COVID-19–induced international travel restrictions were in place in Nigeria, was in marked contrast to reported masses for March–September in 2019 (34,535 kg) and 2021 (12,148 kg). The COVID restrictions on international travel affected land borders, seaport, and airports, with ships only allowing to dock at any Nigerian port after at least 14 days at sea, and may have hampered Nigeria-linked pangolin trafficking activities; however, anti-trafficking measures may also have been reduced over this period. Moreover, any downward trend in confiscated mass in 2020 does not imply that pangolin poaching and/or hunting reduced in 2020. Although we did not investigate local exploitation of pangolins, we suspect derivatives were stockpiled awaiting the full resumption of international travel. The likelihood of stockpiling, however, presents a unique opportunity for increased confiscations and arrests of traffickers through intelligence-gathering before derivatives are shipped out of Africa.

Our MNI estimates are more robust than previous attempts or methods to quantify the number of pangolins contributing to seizure (Challender et al., 2015, Challender et al., 2020; Ingram et al., 2019; Ullmann et al., 2019, 2019; UNODC, 2020a). Estimates from these studies lacked at least one of a) appropriate conversion factors for all the species involved; b) information on the relative contribution of each species to the seizures, and; c) confidence intervals of the estimates. From our estimates, white-bellied pangolins represented 90% of approximately 0.8 million pangolins involved in reported Nigeria-linked seizures. The white-bellied pangolin's disproportionate representation could be attributed to their wide occurrence; they occur in eight of the nine implicated African countries. Additionally, they are perhaps the most common African pangolin (Pietersen et al., 2019) and are frequently hunted and sold in wild meat markets across their range, with an estimated 0.4 million pangolins harvested annually in the Congo basin alone (Ingram et al., 2018). The possible presence of Temminck's pangolin scales in the sorted scale data (aggregated during analysis) was unexpected as none of the incidents in the seizure datasets we used included a shipment destined to or transiting through Nigeria from a Temminck's pangolin range country. The lack of information on local trade routes and sites where the seized pangolin derivatives were harvested are possible explanations for the mismatch. However, our MNI estimates should still be interpreted with caution. It is unlikely that a) all the involved pangolins were killed solely for international trade and; b) all the killed pangolins were harvested from Nigeria alone. Pangolins are harvested for food across Africa (Soewu et al., 2020), and we showed that Nigeria played transit roles in some cases, so pangolins were probably harvested at varying numbers from other African countries. Critically, our estimates only represent a fraction of the trade that was detected, intercepted, and reported. Detected wildlife trafficking incidents probably represent a negligible portion of the overall trade (see also Phelps and Webb, 2015). Three wildlife trade experts provided rough estimates of detected wildlife seizures to be between 2 and 30% of the overall illegal trade [2–10% (B. Brock, pers. comms.); 10% (S. Wasser, pers. comms.); 10–30% (J. Viollaz, pers. comms.)]. The lack of formal pangolin population estimates across Africa limited further investigation into the effect of our MNI estimates on their populations. However, the extraction of hundreds of thousands of individuals in just one decade seems almost certain to be detrimental to the survival of pangolins in Africa.

## 5 Conclusions

We collated and analysed three distinct data types to describe and quantify Nigeria's involvement in trans-national pangolin trafficking. We estimated a minimum of approximately 799,300 pangolins in reported Nigeria-linked seizures from January 2010 to September 2021. This estimate is loosely comparable to a recent global estimate of 895,000 pangolin individuals over 19 years (Challender et al., 2020), suggesting gross underestimation of the scale of pangolin trafficking which could have translated into mismatching anti-trafficking policies. Our study demonstrates the complexity of the global illegal pangolin trade and amplifies the need for concerted conservation efforts at local, national, and international levels to combat pangolin poaching and/or hunting and trafficking. Combining quantitative and qualitative data enhanced our understanding of Nigeria's role in pangolin trafficking and aided our interpretation of the study's overall findings. We thus advocate for the use of a similar multi-pronged approach elsewhere in illegal wildlife trade research, integrating direct data on the species of interest together with information from important stakeholders. Fundamentally, our findings underscore Nigeria's key role to combat global pangolin trafficking and provide important information to support law enforcement efforts to curb the global illegal pangolin trade. We recommend the following based on our findings. First, NCS (and similar enforcement agencies in other pangolin range countries) should, in its mandate, prioritise detection and interception of illegally traded wildlife derivatives as well as training of its law enforcement personnel in illegal wildlife trade detection and documentation. Second, pangolin range countries should take active steps to monitor the trade and ensure proper documentation of seizures. Establishing and regularly updating a national seizure database would be useful next steps. Third, wildlife laws in pangolin range countries must be fully enforced, with emphasis placed on apprehending and prosecuting traffickers, which as shown by Shepherd et al. (2017), will deter other traffickers. Our study has shown that CITES listings alone do not halt trafficking. Fourth, more research should be dedicated to understanding pangolin scale sourcing and supply chains, and in particular the relative roles of the commercial trade and local consumption in motivating pangolin poaching and/or hunting (see Ingram et al., 2021). Fifth, governments of implicated African countries - especially Nigeria - should strengthen law enforcement efforts at key seaports (Appendix B), invest in inspection equipment such as scanners and sniffer dogs, increase the number of inspection staff at borders, and prioritise intelligence-gathering operations (in collaboration with relevant national agencies and non-government organisations). Implementing these measures has the immediate benefit of facilitating the confiscations of possibly stockpiled shipments waiting to be trafficked.

## Funding

This work was supported by the Bill & Melinda Gates Foundation [OPP1144]. This work was also supported by the British High Commission in Nigeria (INT 2021/NIA C19 01) and Wildlife Conservation Network (through WCN-WCS Joint Scholarship for Wildlife Conservation awarded to CAE). We also acknowledge funding from the UK Research and Innovation’s Global Challenges Research Fund (UKRI GCRF) through the Trade, Development and the Environment Hub project (project number ES/S008160/1). DJI and LC are supported by the United States 10.13039/100000202Fish and Wildlife Service and USAID.

## CRediT authorship contribution statement

**Charles A. Emogor**: Conceptualization, Methodology, Investigation, Formal analysis, Writing - Original draft, Funding acquisition. **Daniel J. Ingram**: Conceptualization, Methodology, Visualization, Formal analysis, Writing - Original draft. **Lauren Coad**: Conceptualization, Methodology, Writing - Original draft, Supervision. **Thomas Worthington**: Visualization, Formal analysis, Writing - Original draft. **Andrew Dunn**: Conceptualization, Writing - Original draft, Funding acquisition. **Inaoyom Imong**: Conceptualization, Writing - Original draft. **Andrew Balmford**: Conceptualization, Methodology, Writing - Original draft, Funding acquisition, Supervision.

## Declaration of competing interest

The authors declare that they have no known competing financial interests or personal relationships that could have appeared to influence the work reported in this paper.

## Figures and Tables

**Table:** 

Species (extant continent)	Total body mass in kg (sample range: number of samples; sex. Country)	Mass-to-Individual Conversion Factor: dried mass of complete scales per individual in g (sample range: number of samples; sex. Country)	Source(s)
Black-bellied pangolin (Africa)	1.07 (1.6–784: n = 3; female. Nigeria)	145 (118–188: n = 3; adult female. Nigeria)^a^	C. Emogor, unpublished data
	0.79 (n = 1; adult male. Nigeria)	126 (n = 1; adult male. Nigeria)	C. Emogor, unpublished data
	–	318.82 (n = 1; adult female. Côte d'Ivoire)^a^	M. Shirley and G. Assovi, unpublished data
	–	313.66 (n = 1; adult male. Côte d'Ivoire)^a^	M. Shirley and G. Assovi, unpublished data
Giant pangolin (Africa)	32.1 (n = 1; male. Uganda)		S. Nixon and N. Matthews, unpublished data
	28.8 (n = 119; unsexed. Gabon)		Mambeya et al., 2018
	–	3600 (n = 1; unsexed)	Tikki Hywood Trust, unpublished data
Temminck's pangolin (Africa)	5.6 (2.5–10.2: n = 28; female. South Africa)	–	D. W. Pieterson, unpublished data (as cited in Hoffmann et al., 2020)
	6.0 (2.6–10.6: n = 50; male. South Africa)	–	D. W. Pieterson, unpublished data (as cited in Hoffmann et al., 2020)
	9.3 (2.5–16.1: n = 29; male. South Africa, Zimbabwe)	–	Coulson, 1989; Heath and Coulson, 1997a; Heath and Coulson, 1997b; Jacobsen et al., 1991; Swart et al., 1999
	9.0 (4.6–15.8: n = 28; female. South Africa, Zimbabwe)	–	Coulson, 1989; Heath and Coulson, 1997a; Heath and Coulson, 1997b; Jacobsen et al., 1991; Swart et al., 1999
	21 (n = 1; Sudan)	–	Sweeney, 1974
	–	2270 (n = 136; South Africa, Zimbabwe, and Sudan)^b^	D. W. Pieterson, unpublished data (as cited in Pietersen et al., 2020); Coulson, 1989; Heath and Coulson, 1997a; Heath and Coulson, 1997b; Jacobsen et al., 1991; Swart et al., 1999; Sweeney, 1974
White-bellied pangolin (Africa)	1.54 (0.84–2.14: n = 9; unsexed. Nigeria)	–	C. Emogor, unpublished data
	2.6 (1.94–2.88: n = 11; female. Nigeria)	–	Kingdon and Hoffmann, 2013
	2.36 (1.74–2.86: n = 4. Nigeria)	–	Kingdon and Hoffmann, 2013
	2.06 (0.84–2.67: n = 3; juvenile male. Nigeria)	192 (120–257: n = 3; 2 adult and 1 juvenile male. Nigeria)^c^	C. Emogor, unpublished data
	1.09 (1.33–0.85: n = 2; adult females. Nigeria)	133.5 (141–126: n = 2; adult females. Nigeria)^c^	C. Emogor, unpublished data
	–	199.32 (152.31–246.33: n = 2; juvenile and adult males, respectively. Côte d'Ivoire)^c^	M. Shirley and G. Assovi, unpublished data
Chinese pangolin (Asia)	4.5 (2.1–8.5: n = 20; male. China)	–	Wu et al., 2004b; S. Wu, unpublished data (as cited in Wu et al., 2020)
	3.5 (2.2–5.7: n = 20; female. China)	–	Wu et al., 2004b; S. Wu, unpublished data (as cited in Wu et al., 2020)
	5 (3.5–7.6: n = 19; female. Taiwan)	–	Chin et al., 2015
	–	573.47 (n = 35)	Zhou et al., 2012
Indian pangolin (Asia)	14.25 (11–19.3: n = 4; male. Pakistan)	–	Irshad et al., 2015; Roberts, 1977
	14.55 (9.1–20: n = 2: female. Pakistan)		Irshad et al., 2015; Roberts, 1977
	10.92 (9.88–12.05: n = 3; male. India)	–	Mohapatra and Panda, 2013
	9.8 (9–10.59: n = 53; female. India)	–	Mohapatra and Panda, 2013
	41.45 (34.15–48.76: n = 2; male. Sri Lanka)	–	Perera et al., 2021
Philippine pangolin (Asia)	4.9 (2.7–7.3: n = 9; male. Philippines)	–	S. Schoppe, unpublished data (as cited in Schoppe et al., 2020)
	3.2 (3–3.5: n = 4; female. Philippines)		S. Schoppe, unpublished data (as cited in Schoppe et al., 2020)
Sunda pangolin (Asia)	5.09 (2.8–9.1: n = 21; male)	–	Save Vietnam's Wildlife, unpublished data (as cited in Chong et al., 2020)
	4.5 (2.9–6.3: n = 21; female)	–	Save Vietnam's Wildlife, unpublished data (as cited in Chong et al., 2020)
	–	360.51 (n = 119)	Zhou et al., 2012

a The average of the presented Mass-to-Individual Conversion Factors was used for black-bellied pangolins (i.e., 198.91 g: n = 6).

b Derived by calculating the dry scale proportion to overall body mass of 26.5% (25–28%; D. Pietersen pers. comms) from the average of the mean total body masses of Temminck's pangolin presented.

c The average of the presented Mass-to-Individual Conversion Factors was used for white-bellied pangolins (i.e., 177.38 g: n = 7).

**Table:** 

Port or inspection location	Country	Number of incidents	Mass (kg)	Port type
Kwai Chung customhouse cargo examination compound	Hong Kong	4	24,568	Seaport
Pasir Panjang export inspection station	Singapore	1	12,900	Seaport
Tien Sa port	Vietnam	2	10,000	Seaport
Tsing Yi cargo examination compound	Hong Kong	2	9900	Seaport
Cai Mep international terminal	Vietnam	1	5264	Seaport
Sepanggar port	Malaysia	1	5000	Seaport
Saigon port	Vietnam	1	3300	Seaport
Hai Phong port	Vietnam	2	3247	Seaport
Shanghai port	China	1	3100	Seaport
Apapa port	Nigeria	2	2760	Seaport
Lach Huyen port	Vietnam	2	2760	Seaport
Quy Nhon port	Vietnam	1	1547	Seaport
Port Harcourt international airport	Nigeria	1	1530	Airport
Noi Bai international airport	Vietnam	2	1507	Airport
VIP Green port	Vietnam	1	1400	Seaport
Dinh Vu port	Vietnam	1	1360	Seaport
Istanbul airport	Turkey	1	803	Airport
Mallam Aminu Kano international airport	Nigeria	1	803	Airport
Douala international airport	Cameroon	1	718	Airport
Samui international airport	Thailand	1	587	Airport

**Table:** 

Incident country	Year	Mass (kg)	Outcome	Suspect details
Nigeria	2010		Confiscation	1 suspect: Nigerian
Nigeria	2011		Confiscation and arrests but case discontinued (suspect was not taken to court)	1 suspect: Chinese
China	2012	4.23	Confiscation	3 passengers (unknown nationalities)
China	2012	50.38	One suspect arrested and convicted to 3 years in prison in Anyuan District Court (sentence suspended) and fined USD 8171	1 suspect: Chinese
Cameroon	2013	80	Confiscation and arrests	3 suspects: 1 Chinese and 2 Cameroonians
China	2014	57.62	Confiscation and prosecution by Ningbo Intermediate People's Court - sentenced to one year in prison (suspended for one year and six months) and fined 10,000 RMB.	1 suspect: Chinese
France	2014	250	Confiscation	
China	2015	2000	Confiscation and arrest	4 suspects (unknown nationalities)
China	2015	83.5	Confiscation and arrests - case under investigation.	10 suspects (unknown nationalities)
Hong Kong	2015	2000	Confiscation	
China	2015	249	Confiscation and arrest	3 suspects (unknown nationalities)
China	2015	25	Confiscation and arrest (1 of 2)	2 suspects (unknown nationalities)
Viet Nam	2015	42.2	Confiscation	
Viet Nam	2015	4000	Confiscation	
Nigeria	2015	52.5	Confiscation	Abandoned seizure
Nigeria	2015	176.3	Confiscation	
China	2015	1814.37	Confiscation	2 suspects (unknown nationalities)
Thailand	2015	587	Confiscation (ongoing investigation)	
Singapore	2015	324	Confiscation	
Kenya	2016	0.5	Confiscation	Abandoned seizure
Netherlands	2016	42	Confiscation	
Netherlands	2016	57	Confiscation	
Nigeria	2016	381	Confiscation and 2 arrests	2 suspects: Chinese nationals
Nigeria	2016	390	Confiscation	
Nigeria	2016	80	Confiscation	
Hong Kong	2016	7300	Confiscation	
China	2016	58	Confiscation	
Hong Kong	2016	0.09	Confiscation and arrest	4 suspects (unknown nationalities)
China	2016	3100	Confiscation and arrest	3 suspects: 1 Chinese, 2 unknown nationalities
Viet Nam	2017	704	Confiscation	
Viet Nam	2017	322	Confiscation	
Hong Kong	2017	7200	Confiscation	
Malaysia	2017	288	Confiscation and 3 arrest	3 suspects (unknown nationalities)
Malaysia	2017	5000	Confiscation and 1 arrest	1 suspect: Malaysian
Cameroon	2017	1050	Confiscation	
Hong Kong	2018	1800	Confiscation	
Cote d'Ivoire	2018	600	Confiscation and 6 arrests	6 suspects: 2 Ivorians, 1 Guinean, 2 Vietnamese, 1 Chinese
Nigeria	2018	2001	Confiscation and 1 arrest	1 suspect: Chinese
Viet Nam	2018	3300	Confiscation	
Nigeria	2018	8492	Confiscation and 1 arrest	1 suspect: Chinese
Hong Kong	2018	2800	Confiscation	
Nigeria	2018	1771	Confiscation	
Nigeria	2018	450	Confiscation	
Democratic Republic of Congo	2018	1798	Confiscation and 4 arrest	4 suspects: 1 Chinese, 3 Nigerians
Nigeria	2018	23 sacks	Confiscation and 1 arrest	1 suspect: Chinese
Viet Nam	2018	334	Confiscation	
Hong Kong	2018	7100	Confiscation	
Nigeria	2018	1003	Confiscation	
Nigeria	2018	738	Confiscation and 1 arrest	1 suspect: Chinese
Cameroon	2018	718	Confiscation and 6 arrest	6 suspects: 5 Cameroonians and 1 Central African
Nigeria	2018	1	Confiscation	2 suspects: Chinese
Viet Nam	2018	803	Confiscation	
Viet Nam	2018	6000	Confiscation	
Hong Kong	2018	0.095	Confiscation and arrest (given a 2-week custodial sentence)	Unknown number of suspects and nationalities
China	2018	6000	Confiscation and 2 arrest	2 suspects (unknown nationalities)
Nigeria	2018	7560.9	Confiscation and 7 arrests	7 suspects (unknown nationalities)
Viet Nam	2018	47	Confiscation	
China	2018	10,560	Confiscation and 18 arrests	18 suspects (unknown nationalities)
Hong Kong	2019	8268	Confiscation and 2 arrests (charged and granted bail)	2 suspects (unknown nationalities)
Viet Nam	2019	1360.78	Confiscation	
Viet Nam	2019	1400	Confiscation	
Cameroon	2019	2500	Confiscation and 4 arrests (arraigned in Bonabéri court)	4 suspects (unknown nationalities)
Singapore	2019	12,900	Confiscation	
Singapore	2019	12,700	Confiscation	
Viet Nam	2019	5264	Confiscation	
Nigeria	2019	500	Confiscation	
Nigeria	2019	671	Confiscation	
Nigeria	2019	100	Confiscation	
Nigeria	2019	1530	Confiscation	1 suspect: Nigerian
Nigeria	2019	200	Confiscation	
China	2019	10,650	Confiscation and 18 arrest	18 suspects (unknown nationalities)
Viet Nam	2019	1547	Confiscation	
Viet Nam	2019	1700	Confiscation	
Nigeria	2020	9504.1	Confiscation	20 suspects (unknown nationalities)
Nigeria	2020	330	Confiscation	
Nigeria	2020	150	Confiscation	
Nigeria	2021	5329	Confiscation, 1 arrest and ongoing prosecution	1 suspect: 1 Nigerian
Nigeria	2021	4000	Confiscation	
Nigeria	2021	7143	Confiscation, 3 arrests and ongoing prosecution	3 suspects: 1 Nigerian, 2 Guineans
Nigeria	2021	1010	Confiscation and 2 arrests (with plans to prosecute)	2 suspects: 1 Nigerian, 1 Burkinabe

## Appendix D Law enforcement interview questions

1. How many enforcement officials are there currently to conduct inspections in the port/border?
2. On average, how many containers are transported through any of the Nigerian seaports/borders weekly/monthly?
3. Can you estimate what percentage of containers are inspected every week/month?
4. In the containers that you inspect, roughly what percentage do you find pangolin scales in?
5. How are routine inspections at ports/borders conducted - what technologies, including sniffer dogs, are used?
6. What has been the role of intelligence in the pangolin seizures made in Nigeria?
7. What happens to suspected pangolin traffickers and confiscated materials when seizures are made?
8a. Has there been any training on illegal wildlife trade issues organized by the Service or wildlife organisations in Nigeria? If yes, how many, when and what training?
8b. Have the number of training courses increased/decreased/stayed the same from 2011-2016?
8c. Have the number of training increased/decreased/stayed the same from 2017-2020?
9a. Have the number of staff available to search containers increased/decreased/stayed the same from 2011-2016?
9b. Have the number of staff available to search containers increased/decreased/stayed the same from 2017-2020?
10a. Have the number of searches increased/decreased/stayed the same from 2011-2016?
10b. Have the number of searches increased/decreased/stayed the same from 2017-2020?
11a. Has what happened to suspected wildlife traffickers changed from 2011-2016?
11b. Has what happened to suspected traffickers changed from 2017-2020?
12a. Has how scales are shipped (volume, goods they are shipped with, how they are hidden) changed from 2011-2016?
12b. Has how scales are shipped (volume, goods they are shipped with, how they are hidden) changed from 2017-2020?
13a. Have levels of awareness of pangolin trafficking increased, decreased or stayed the same within the NCS from 2010–2016?
13b. Have levels of awareness of pangolin trafficking increased, decreased or stayed the same within the NCS from 2017–2020?

## Appendix E Supplementary data

Image 1
